# Supplementary material for: Whole-Genome Analysis of Mycobacterium neoaurum DSM 1381 and the Validation of Two Key Enzymes Affecting C22 Steroid Intermediates in Sterol Metabolism
Source: Int J Mol Sci. 2023 Mar 24;24(7):6148. doi: 10.3390/ijms24076148 (PMC10094492; doi:10.3390/ijms24076148)
Supplement: Supplementary file 1 [file ijms-24-06148-s001.zip › ijms-2183374-supplementary.pdf]

Table S1. Polymorphisms in gene sequences of *M. neoaurum* DSM 1381.

Description of Gene Product	Gene ID		SNV		aa mutation	
	DSM 43536	DSM 1381	DSM 43536	DSM 1381	DSM 43536	DSM 1381
**Nonsynonymous SNV**						
KshA1	807	14	517A	517G	173Asn	173Asp
Hsd4A	775	46	511A	511G	171Lys	171Glu
ribonuclease	446	372	152T	152A	51Ile	51Asn
oxidoreductase FmdB family transcriptional regulator	441	377	26T	32C	9Leu	11Pro
oxidoreductase	328	485	159C	159G	53Try	53TAG
ferrochelatase	894	878	49T;50C	49C;50A	17Ser	17His
dihydroxy-acid dehydratase	984	965	321G	321C	107Met	107Ile
MFS transporter	1005	986	120A	120G	40Ile	40Met
HNH endonuclease	1309	1278	512A;1234C	512C;1234A	171Lys;412Pro	171Thr;412Thr
NAD(P)/FAD-dependent oxidoreductase	1328	1296	1025C	1025G	342Ala	342Gly
MMPL family transporter	1490	1454	2900C	2900T	967Ser	967Leu
D-glycero-beta-D-manno-heptose 1-phosphate adenylyltransferase	1739	2086	950T	950G	317Leu	317Trp
peptidase M4, thermolysin	1875	1953	3781A	3781G	1261Ile	1261Val
FadR family transcriptional regulator	2235	2188	446T	446A	149Leu	149Gln
DUF4192 domain-containing protein	2366	2319	587C	587G	196Ala	196Gly
hypothetical protein	3271	3077	275C; 278T	245T; 248C	82Ser;83Phe	82Phe;83Ser
LytR family transcriptional regulator	3572	3454	77G	77A	26Arg	26His
UPF0182 family protein	3671	3549	883C	883G	295Arg	295Gly
thioredoxin-dependent thiolperoxidase	3744	3654	230A	230G	77Lys	77Arg

Description of Gene Product	Gene ID		SNV		aa mutation	
	**DSM 43536**	**DSM 1381**	**DSM 43536**	**DSM 1381**	**DSM 43536**	**DSM 1381**
extracellular solute-binding protein	3937	3761	1034T	1034G	345Val	345Gly
MBL fold metallo-hydrolase	4053	4069	751C	700A	234Pro	234Thr
sensor histidine kinase	4084	4099	421C	421G	141His	141Asp
agmatine dei minase family protein	4341	4344	250G	250A	84Asp	84Asn
virulence factor *mce* family protein	4358	4329	944T	962C	321Val	321Ala
HNH endonuclease	5068	3200	326C;380C	326G;380A	109Ala;127Thr	109Gly;127Lys
alpha/beta hydrolase/acetyl esterase/lipase	5160	5044	154G	154A	52Glu	52Lys
YVTN beta-propeller repeat-containing protein	1875	1953	4201A	3781G	1261Ile	1261Val
SDR family NAD(P)-dependent oxidoreductase	1151	1123	upstream 34G	upstream 34T		
**Synonymous SNV**						
hypothetical protein	802	59	3138C	3138T	1046Phe	1046Phe
MogA/MoaB family molybdenum cofactor biosynthesis protein	447	371	474C	474T	158Ile	158Ile
hypothetical protein	208	602	1140C	1140T	380Arg	380Arg
apolipoprotein N-acyltransferase	1068	1047	1272C	1272T	424Ile	424Ile
cupin domain-containing protein	1560	1522	360C	360T	120Phe	120Phe
4-hydroxy-2-oxopentanoic acid aldolase	1271	1243	327G	327C	109Arg	109Arg
3-ketoacyl-(acyl-carrier-protein) reductase	4311	4374	549G	549A	183Leu	183Leu
MMPL family transporter	4817	4835	723C	723T	241Phe	241Phe
glutamate synthase large subunit	5263	5153	3156G	3156A	1052Gln	1052Gln
benzoylsuccinyl-CoA thiolase	5338	5225;5228	255G;256T	255A;256C	85Glu;86Leu	85Glu;86Leu

Table S2. Gaps/Inserts in gene sequences of *M. neoaurum* DSM 1381.

Indels	DSM 43536	DSM 1381	Indels in DSM 1381	Mutation start position
**Coding region**				
acyltransferase	187	624	gap, 1bp	461
type I polyketide synthase	905-906	888	insert, 802bp	1434
YVTN beta-propeller repeat-containing protein	1875	1953	gap, 324bp	2262
inner membrane protein YhjD	2089-2090	1741	gap, 40bp	558
2,4-dienoyl-CoA reductase	2435	2389	insert, 4bp	660
peptidyl-prolyl cis-trans isomerase	2469	2422	gap, 36bp	613
acyl-CoA synthetase	2617-2616	2573	gap, 34bp	807
Rne/Rng family ribonuclease	2828	2905	insert, 15bp	2874
hypothetical protein	3137	3380	gap, 64bp	423
hypothetical protein	3837	3857	gap, 39bp	315
hypothetical protein	3937	3761	insert, 20bp	1054
alcohol dehydrogenase	4085	4101	insert, 1bp	989
HNH endonuclease	4402	4285	gap, 53bp	79
HNH endonuclease	4563-4564	4605	gap, 47bp	1205
HNH endonuclease	5002	3922	gap, 58bp	200
membrane protein	5079	3189	gap, 38bp	366
methyltransferase	5153	5051	gap, 2bp	37
**Non-coding region**				
Inactive dihydropteroate synthase 2; glucosyl-3-phosphoglycerate synthase	130-131	679-678	gap, 103bp	upstream,downstream
coumarate CoA ligase family protein; methionine synthase	1595-1593	1557-1555	gap, 36bp	downstream, downstream

Indels	DSM 43536	DSM 1381	Indels in DSM 1381	Mutation start position
PucR family transcriptional regulator; 2-nitropropane dioxygenase	2092-2093	1738-1739	gap, 36bp	downstream, downstream
NAD(P)H-quinone dehydrogenase; gamMa-glutamyl cyclotransferase	2132-2133	1698-1699	gap, 35bp	upstream, upstream
4a-hydroxytetrahydrobiopterin dehydratase; proline--tRNA ligase	2247-2248	2200-2201	gap, 33bp	downstream, upstream
hypothetical protein; VWA domain-containing protein-4102	4086-4085	4102-4101	gap, 33bp	downstream, upstream
MinD/ParA family protein; DUF4229 domain-containing protein	4148-4149	4161-4162	gap, 43bp; gap, 13bp	downstream, upstream
RDD family protein; hypothetical protein	3402-3403	4713-4714	gap, 66bp	upstream,downstream

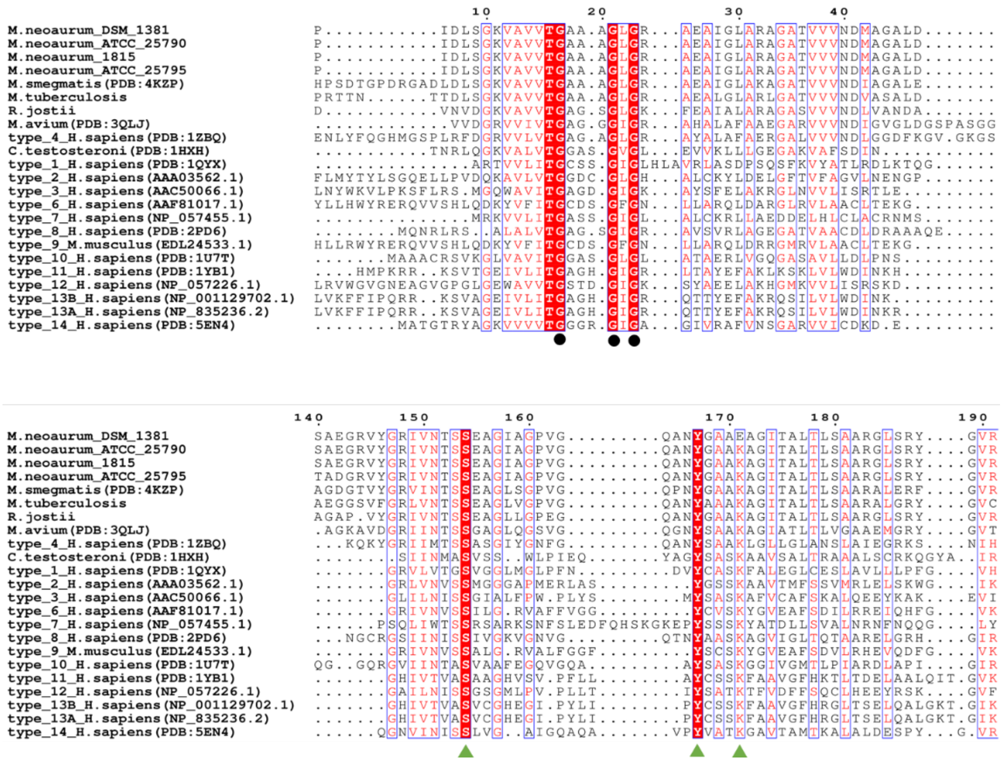

Figure S1. Alignment of Hsh4A homologs amino acid sequence.

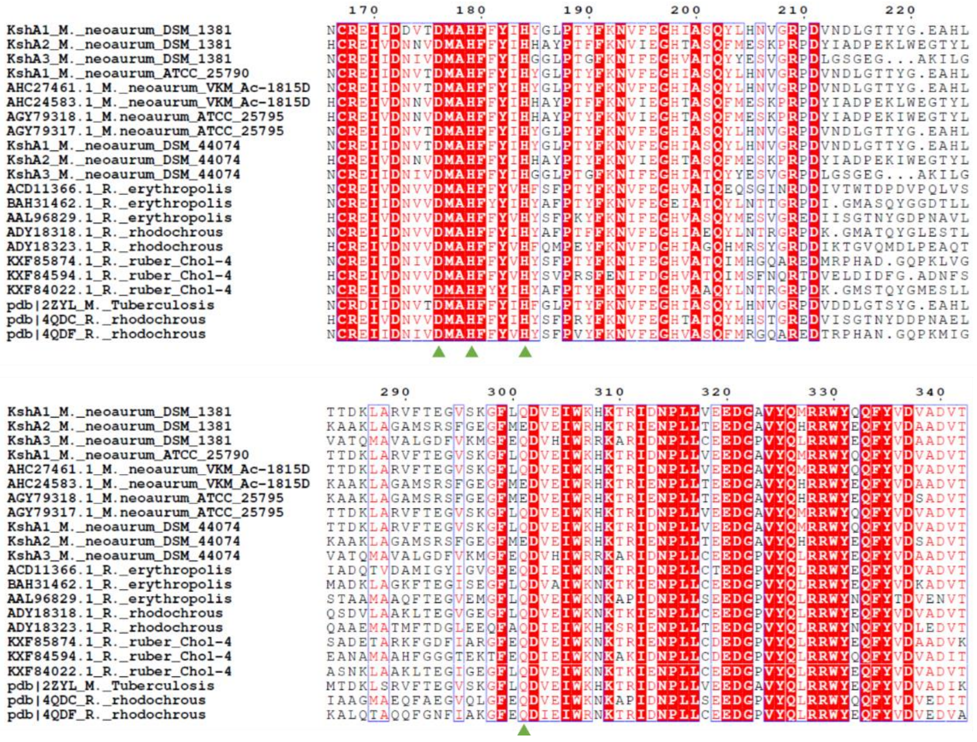

Figure S2. Alignment of KshA homologs amino acid sequence.
